# Interfaces in reinforced epoxy resins: from molecular scale understanding towards mechanical properties

**DOI:** 10.1007/s00894-023-05654-w

**Published:** 2023-07-12

**Authors:** Julian Konrad, Dirk Zahn

**Affiliations:** Lehrstuhl für Theoretische Chemie/Computer Chemie Centrum, Friedrich-Alexander Universität Erlangen-Nürnberg, Nägelsbachstraße 25, 91052 Erlangen, Germany

**Keywords:** Epoxy resins, Reinforced polymers, Molecular simulation

## Abstract

**Context:**

We report on atomic level of detail analyses of polymer composite models featuring epoxy resin interfaces to silica, iron oxide, and cellulose layers. Using “reactive” molecular dynamics simulations to explore epoxy network formation, resin hardening is investigated in an unprejudiced manner. This allows the detailed characterization of salt-bridges and hydrogen bonds at the interfaces. Moreover, our sandwich-type composite systems are subjected to tensile testing along the interface normal. To elucidate the role of relaxation processes, we contrast (i) direct dissociation of the epoxy-metal oxide/cellulose contact layer, (ii) constant strain-rate molecular dynamics studies featuring (visco-)elastic deformation and bond rupture of the epoxy resin, and (iii) extrapolated relaxation dynamics mimicking quasi-static conditions. While the fracture mechanism is clearly identified as interface dissociation of the composite constituents, we still find damaging of the nearby polymer phase. The observed plastic deformation and local cavitation are rationalized from the comparably large stress required for the dissociation of salt-bridges, hydrogen bonds, and van der Waals contacts. Indeed, the delamination of the contact layers of epoxy resins with slabs of silica, magnetite, and cellulose call for a maximum stress of 33, 26, and 21 MPa, respectively, as compared to 84 MPa required for bulk epoxy yielding.

**Methods:**

Molecular dynamics simulations using the Large-scale Atomic/Molecular Massively Parallel Simulator (LAMMPS) code were augmented by a Monte Carlo–type procedure to probe epoxy bond formation (*Macromolecules* 53(22): 9698–9705). The underlying interaction models are split into conventional Generalized Amber Force Fields (GAFF) for non-reacting moieties and a recently developed reactive molecular mechanics potential enabling epoxy bond formation and cleavage (*ACS Polymers Au* 1(3): 165–174).

## Introduction

Industrial needs for resilient materials of light-weight and low-cost are often addressed by composites based on epoxy resins [1]. This thermosetting polymer is formed by addition reactions of epoxy and linker moieties, such as bisphenol-F-diglycidyl ether and 2-methylbenzene-1,3-diamine, respectively. Using stoichiometric compositions of the precursors, nearly 100% conversion may be achieved. Consequently, the multinary amine used as the linker will establish multiple (for the present example 4) connections and act as a node within a 3-dimensional network of the polymer.

In the presence of additional particles, fibers or substrate surfaces, the epoxy-linker curing reactions need to adapt to the corresponding boundaries—and (covalent) polymer network formation is altered by hydrogen bonding, salt-bridges, etc. at the interface. The latter may be sufficiently strong to enable the use of epoxy resins as adhesives and surface coatings [2]. Upon full immersion of the foreign phases into the resin, particle- or fiber-based epoxy composites are obtained, and a range of polymer reinforcement mechanisms may be achieved. On such basis, state-of-the-art epoxy composite formulations even combine a series of favorable effects such as toughening, shock resistance, and abrasion behavior [3, 4].

While a large body of experimental research and industrial development efforts is dedicated to the empirical engineering of constituents and processing parameters, in-depth understanding is increasingly collected from atomic force and electron microscopy [5–7]. Likewise, modelling and simulation efforts of epoxy resin and their composites range from typically empirical formulations of constitutive models and continuum mechanics approaches to atomic-scale techniques based on quantum chemistry and molecular mechanics [8–10].

For the interpretation of polymer toughening from fiber and/or particle incorporation, the commonly discussed aspects range from frontal wakes and bridging effects in cracks to larger scale crack pattern formation [11]. These concepts mainly address the micrometer scale structure of resin composites, a particularly evident example for this being crack deflection within an array of embedded particles or fibers. In terms of modelling and simulations, it is thus intuitive to suggest meso-scale approaches such as finite element techniques to analyze the mechanisms of crack propagation in reinforced epoxy resins.

However, in lack of detailed information of the molecular scale processes at polymer-fiber/particle interfaces, these approaches often rely on ad hoc assumptions and empirical parameterization. To this end, much (if not all) experimental information such as stress-strain diagrams is needed for model development, whereas rigorous benchmarking is often limited to checks of self-consistency. An appealing alternative to such semi-empirical materials modelling could be given from atomic simulations. The latter are typically based on molecular mechanics that coarsen atomic interaction forces as observed from first principles calculations. Thanks to quantum chemistry, we may hence employ atomistic simulation data as a non-empiric route to assessing the inputs needed for meso-scale particle or continuum models [12–15].

In what follows, we exemplify this strategy by characterizing the interaction forces governing interfaces of epoxy resins to commonly used composite constituents and substrate materials. Along this line, we account for silica as prominent species for particle incorporation, whereas cellulose reflects an important compound for polymer reinforcement from fibers, respectively. In turn, iron oxide, here magnetite, is addressed because of its importance for steel surfaces (mimicking corrosion passivation from bluing). Apart from atomic-scale analyses, a key focus is dedicated to the critical stress and mechanical work needed for interface separation—which is suggested as direct inputs for coarse-grained particle concepts and continuum models.

## Simulation details

The silica-epoxy (i), magnetite-epoxy (ii), and cellulose-epoxy (iii) interfaces are described as sandwich models featuring 2D periodic slabs of the two constituents that are staggered using 3D periodic boundary conditions. As a starting point to model system, (i) we employ an amorphous silica model developed in an earlier study [15]. It reflects a 7.2×7.6 nm^2^ sized slab of 1.8-nm thickness and comprises 3024 Si, 5799 O^2-^, and 498 OH^-^ ions, respectively; details on model preparation from melt quenching are provided in refs. [12, 15]. The hydroxide ions are located at the outer boundaries of the slab surface and account for the protonation state of silica subjected to humidity at neutral pH (4.6 OH per nm^2^) [16]. While experiments suggest −0.1 to −0.5 e/nm^2^ surface charge density at pH=7 [17–20], in our models we however impose zero charge to ensure numerical stability of the 2D periodic slab model.

In turn, (ii) the iron oxide slab was constructed from the unit cell replicated 7 × 7 × 1.7 to a super cell of magnetite with (100) surface. In analogy to the silica surface, also the outermost O^2-^ ions of the magnetite slab were protonated (while removing iron ions from the surface to maintain charge neutrality) to mimic the effect of air humidity. While the explicit surface structure of humid magnetite surfaces is still under debate [21], our model complies with the current mechanistic picture of charge neutralization from subsurface iron vacancies. The cation vacancies were randomly assigned in the outermost Fe layers and the entire slab model was allowed to relax from 5-ns scale molecular dynamics runs. The resulting 5.7×5.7 nm^2^ slab features a thickness of 1.3 nm and comprises 700 Fe^2+^, 1300 Fe^3+^, 2500 O^2-^, and 300 OH^-^ ions, respectively.

The third model (iii) mimics a cellulose slab formed by 5 stacks of cellulose sheets—each of which featuring 7 parallel cellulose fibers. The nanometer scale fiber models were described by oligomer chains of 11 covalently connected, i.e., β(1-4) linked, D-glucose molecules. Upon geometry optimization from energy minimization, fiber-fiber hydrogen bonding leads to a well-defined 6.0×6.0 nm^2^ slab of 1.8-nm thickness.

All three slab models were first relaxed in vacuum to achieve well-defined 2D-periodic layers. Next, the simulation cells were fixed in two dimensions according to the 7.2×7.6 nm^2^, 5.7×5.7 nm^2^, and 6.0×6.0 nm^2^ sized layer segments of models (i), (ii), and (iii), respectively, whereas the polymer precursors are added along the remaining dimension. For each system, 1024 EPON (bisphenol-F-diglycidyl ether) and 512 DETDA (4,6-diethyl-2-methylbenzene-1,3-diamine) molecules were introduced and 3D-periodic boundary conditions are applied to create sandwich-style bulk systems.

Benefitting from our earlier development of a reactive molecular mechanics model for describing the polymerization reaction [13], we explored epoxy resin curing in the presence of the 3 different interface types. For the sake of comparability, the entire simulation protocol for molecular dynamics simulation of epoxy network formation is adopted from refs. [13, 22]. While this reference is based on the formation of the 3D-periodic bulk epoxy resin, for the present study independent polymerization runs were performed for each of the interface models. Consequently, our sandwich models do not reflect simplistic stacks of two constituents placed next to each other, but provide a much more realistic account of composite resins. Indeed, the explicit modelling of polymerization reactions in the presence of the metal oxide or cellulose layers is crucial to allow structural relaxation of both composite constituents including the alignment of epoxy strands near the interfaces.

The atomic interactions of the EPON, DETDA, and hardened epoxy networks are described by the reactive molecular mechanics model reported in ref. [13]. Therein, the OPLS-AA force-field [23] was extended by additional short-range potentials that mimic the addition reaction of epoxy and amine moieties. Likewise, the OPLS-AA model is used for the epoxy-metal oxide, epoxy-cellulose, and cellulose-cellulose interactions, respectively. To account for the specific nature of the metal-oxide bonding, tailor-made potentials using more sophisticated pair potentials for silica [12] and magnetite [24–26] were adopted from the literature.

The van der Waals pair potentials were cut off at a distance delimiter of 1.2 nm. In turn, the more long-rage Coulomb interactions were described by the damped-shifted force approach [27]. For this, shifting from real-space to k-space was implemented at a cut-off distance of 1 nm and a damping factor of 0.1 Å^−1^—as suggested by Fennel [28]. The epoxy curing runs are based on Monte Carlo attempts of polymer linking reactions, combined with molecular dynamics runs for relaxation. In full analogy to our previous study of bulk epoxy curing, each linking attempt is characterized from 5-ns relaxation runs at 460 K and 1 atm, respectively, whereas full polymerization is assumed after the acceptance rate of linking attempts reduces to < 0.1 % [13]. The constant-temperature, constant-pressure molecular dynamics simulations were performed with a timestep of 0.5 fs and the LAMMPS package [29]. Using the built-in Nosé-Hoover algorithm, good decoupling of thermostat and barostat fluctuations was ensured from choosing the corresponding relaxation times as 0.1 ps and 1.0 ps, respectively.

The separation of the composite interfaces is explored by two rather dissimilar strategies. We firstly explored (A) block-wise separation of the two entities without structural relaxation. This block-wise separation mimics the idealized case of brittle fracture by separation of the contact layers of the sandwich models. Thus, we argue that this “brute-force” approach refers to an upper estimate of composite fragmentation at extremely high speed. In turn, tensile testing is also explored (B) by an iterative procedure of gradual displacement steps. In each step, we move the two epoxy blocks embedding the metal oxide or cellulose slabs by shifting all atoms collectively by Δ*z* (upper epoxy block) and −Δ*z* for the lower epoxy block of the sandwich models. Consequently, the cell vector along interface normal (*z* direction) of the 3D-periodic simulation models are elongated by 2∙Δ*z*. For this, cell vector elongation is implemented at a 0.015, 0.15, and 1.5 m/s using three independent molecular dynamics runs, respectively. The temperature is kept at 300 K—which allows relaxation as governed by the crossing of energy barriers to epoxy reorganization [30]. To avoid artifacts from pulling along the *z*-direction, the thermostat is only applied to velocities along *x* and *y* directions, respectively.

On this basis, run (A) is confined to block-wise separation, whereas only run (B) enables visco-elastic relaxation and gradual bond rupture in the epoxy resin. Subsequently, we used our slowest deformation runs (B) for $\dot{s}$ = 0.015 m/s to perform additional relaxation analyses. For this, selected snapshots from the tensile testing were subjected to constant volume runs. Based on 10-ns scale stress relaxation profiles, exponential fits are employed to predict the residual stress upon infinite relaxation time. This provides extrapolation to the quasi-static tensile testing at vanishing strain rate.

To compare the resulting fracture pathways, we monitor the stress *σ* along the pulling direction *z*—which as directly calculated as the virial *σ*_*zz*_, which only reflects atomic interaction forces directed along *z*. Moreover, the work of separation is calculated from the integral of the stress profiles *σ*_*zz*_(*s*) as functions of the sandwich separation by *s* = Σ Δ*z*.

## Results

### Epoxy curing in the presence of interfaces: model preparation

The realistic account of epoxy composite interfaces calls for the explicit consideration of the formation process of the material. Akin to the experimental preparation of such composites, also simulation models should not be based on simply attaching blocks of cured polymer to other constituents. Instead, the guest constituents are typically introduced to the epoxy/hardener liquid mixture before or at the very beginning of the polymerization reactions. Thus, polymer network formation occurs in the direct presence of filler particles or fibers.

To ensure the unbiased investigation of the interplay of polymer-polymer interactions and the forces of the polymer-metal oxide/cellulose interfaces all sandwich models were subjected to a combined Monte Carlo/molecular dynamics simulation procedure of gradual network formation and relaxation [13]. To this end, we adopt the epoxy-silica and the epoxy-cellulose sandwich systems from our earlier study reported in ref. [31]. In turn, the epoxy-magnetite sandwich model was set up in an analogous manner, fully adopting the simulation protocol to ensure full comparability of all three sandwich systems.

In brief, relaxation of the interfaces and the unreacted EPON/DETDA precursor liquid is allowed from 2.5-ns scale molecular dynamics simulation runs at 1 atm and 460 K using the conventional, non-reactive force-fields. Next, we switched to our reactive force field [13] within an additional 5-ns run. Within such short time scales, only about 80% curing can be achieved. However, to mimic longer-termed polymerization and relaxation of the sandwich models, a combined MC/MD approach [13, 30] is employed until the degree of crosslinking converged. This lead to epoxy curing at a degree of 96, 96, and 95 % for the interface systems featuring the silica, cellulose, and magnetite slabs, respectively. While polymerization is explored at 460 K, in preparation of the tensile testing at ambient conditions, all three sandwich systems were subjected to further relaxation runs of 100 ns at 300 K and 1 atm. All structural and mechanical data discussed in the following was collected at 300 K.

**Fig. 1:**
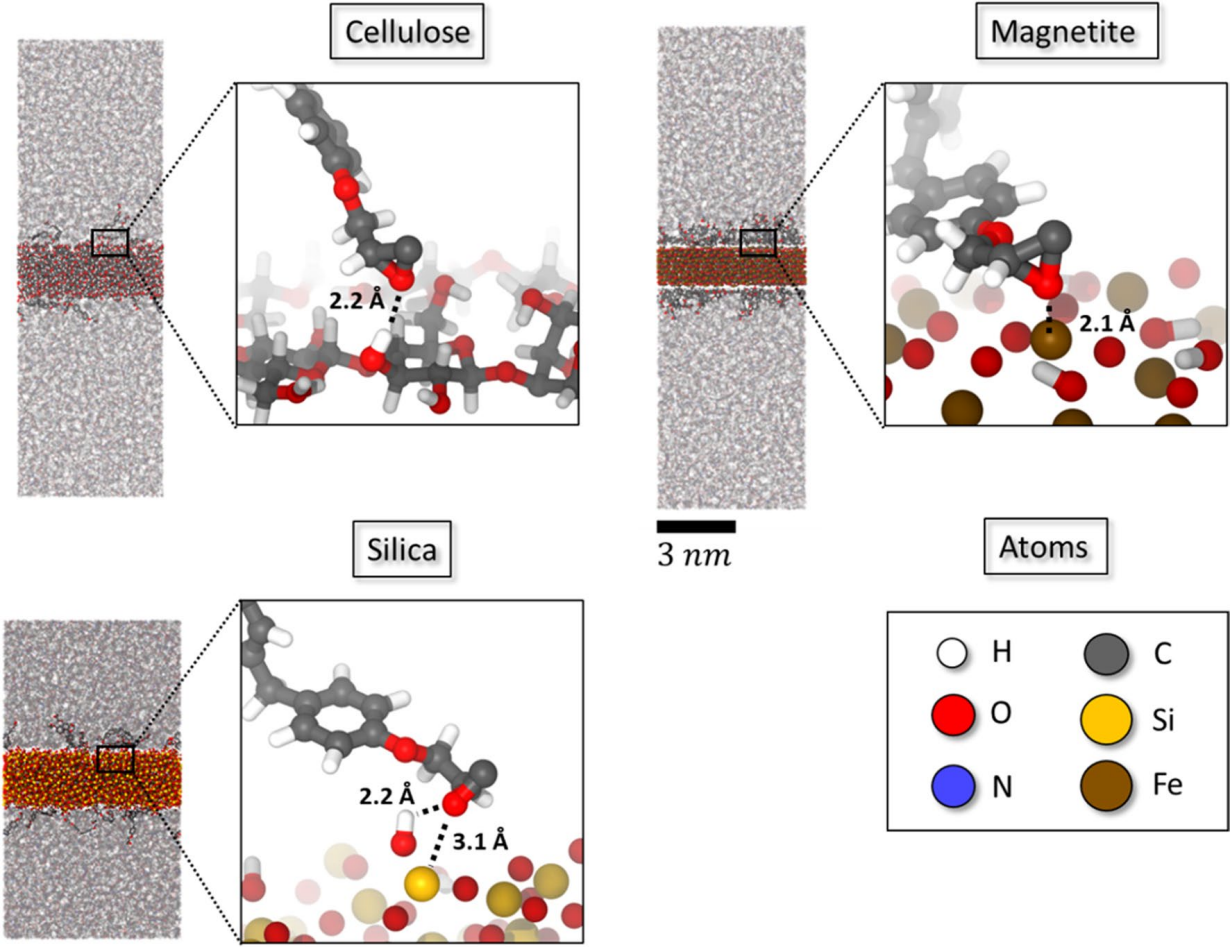
Illustrations of the sandwich-type models featuring interfaces of the epoxy resin to cellulose, silica, and magnetite layers, respectively. Each model is subjected to 3D-period boundary conditions and was created in an unbiased manner by firstly placing a stoichiometric mixture of unreacted EPON and DETDA species and then exploring network formation from a combined Monte Carlo/molecular dynamics simulation approach [31, 32]

Illustrations of the fully relaxed sandwich models are shown in Fig. 1. At the interface, polar entities of the polymer network are observed to form hydrogen bonds and salt-bridges with the metal oxide and cellulose slabs. Among the three sandwich models, the cellulose-epoxy interface displays the weakest binding. Indeed, only a small number of hydrogen bonds between the hydroxyl groups of cellulose and the epoxy oxygen atoms were observed. These amount to a hydrogen bond density of 0.17 per nm^2^, whereas other binding motifs appear negligible. The underlying O-H distances were found in the range of 0.2–0.25 nm thus indicating rather weak hydrogen bonds. For comparison, the hydrogen bonds within the uppermost cellulose layer show O-H distances of 0.12–0.15 nm and the overall density of cellulose-cellulose hydrogen bonds amounts to 1.3 per nm^2^, respectively.

In turn, the magnetite-epoxy interactions are clearly dominated by Fe-O and Fe-N salt-bridges at occurrences of 0.86 and 0.02 contacts per square nanometer, respectively. Practically no hydrogen bonding between the polymer and the (surface-hydroxylated) Fe_3_O_4_ slab is observed. The underlying Fe-O and Fe-N distances of 0.18–0.32 nm however indicate strong electrostatic interactions. Finally, the silica-epoxy interface features Si-O (but no Si-N) salt-bridges at an occurrence of 0.22 contacts per square nanometer and distances of 0.23–0.32 nm, in parallel to hydrogen bonding donated by the silanol groups to O (0.24 per nm^2^) and N (0.05 per nm^2^) acceptors of the polymer, respectively. While representative structural motifs are highlighted in Fig. 1, a more quantitative assessment of the interfacial adhesion forces is elaborated from the tensile testing studies as discussed in the following.

### Epoxy-based composites under mechanical load: tensile testing

Two types of molecular dynamics runs were performed to elucidate interface separation. In run A, we scanned the separation energy by block-wise displacement of the polymer domains and the cellulose/metal oxide slabs. To provide an upper estimate of rapid interface dissociation, in run A no structural relaxation was performed and energy profiles as functions of the displacement *s* were instead taken from a series of single point calculations. In turn, the derivatives of these energy profiles indicate the adhesive forces of the interfaces. While this procedure offers very rapid assessment of overall interface energies, the mechanisms of epoxy-cellulose and epoxy-metal oxide dissociation are surely more complex. Indeed, run A reflects the limiting case of *concerted* salt-bridge and hydrogen bond cleavage that gives rise to rather unrealistic estimates of the maximum stress *σ*_max_.

**Fig. 2:**
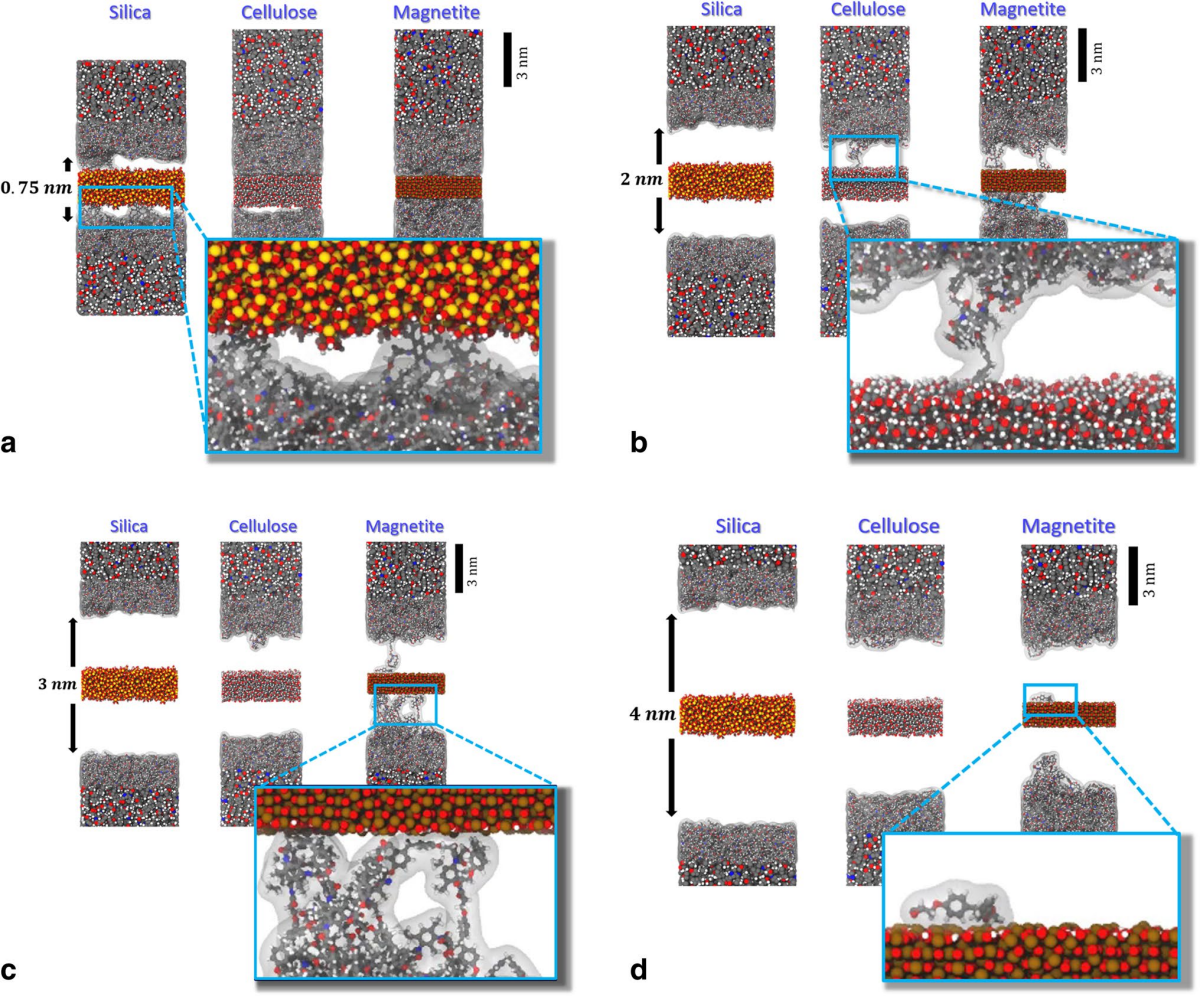
Snapshots of the three composite models during tensile testing. The atom colors are chosen analogous to Fig. 1. Bold spheres indicate atomic species which were held fixed in order to implement separation of the polymer blocks and the cellulose/metal oxide slabs of our sandwich models. **a** Upon separation by *s* = 0.75 nm, we find substantial dissociation of the epoxy-silica (left) and epoxy-cellulose (center) interfaces, whereas the magnetite-epoxy interface (right) features (visco)-elastic deformation rather than hydrogen bond/salt-bridge dissociation. **b** Upon separation by *s* = 2 nm, we find full separation of the epoxy-silica interface, whereas the cellulose-epoxy and magnetite-epoxy interfaces abandon hydrogen bonds and salt-bridges more gradually. **c** Upon separation by *s* = 3 nm, the magnetite-epoxy interface (right) shows strong plastic deformation of the epoxy network during a cascade of salt-bridge dissociation events. **d** Upon full separation (*s* = 4 nm or larger), the epoxy block shows pronounced plastic deformation. The particularly strong salt-bridges of the epoxy-magnetite interface provoked the dissociation of a single EPON molecular fragment from the epoxy network (close-up at the lower right)

To go beyond concerted bond dissociation, and thus enable *proper relaxation* of the sandwich models during tensile testing, we performed nanosecond scale molecular dynamics simulation in run B. For this, we implemented epoxy and cellulose/metal oxide separation at constant deformation rate $\dot{s}$. In parallel runs, a series of $\dot{s}$ = 0.015, 0.15, and 1.5 m/s was explored to elucidate the effect of limited (yet non-zero) relaxation times. This series is also in line with the tensile deformation rates used in our earlier study on bulk epoxy testing as reported in ref. [13]. In Fig. 2, snapshots taken from the tensile testing run B are illustrated for all three sandwich models.

Before the dissociation of hydrogen bonds and salt-bridges at the interfaces, the epoxy resin features elastic and visco-elastic deformation. Upon gradual release of interfacial bonds, also the polymer network experiences significant plastic deformation. This particularly holds for the magnetite-epoxy sandwich model. As a consequence, we do not observe abrupt decline of the adhesion at the interface, but a more complex interplay of polymer network rearrangement and step-wise dissociation of hydrogen bonds and salt-bridges. The latter process extends over nanometer scale displacements of the composite constituents. While our sandwich models show maximal stress at *s*(*σ*_max_) = 0.16–0.26 nm, full interface dissociation is observed at separation by more than 1 nm.

**Fig. 3:**
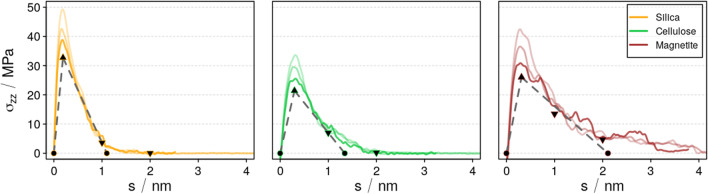
Profiles of tensile stress *σ*_*zz*_ as a function of the separation *s* applied to interfaces of the epoxy resin and (left to right) silica, cellulose, and magnetite, respectively. Data assessed at strain rates of $\dot{s}$ = 0.015, 0.15, and 1.5 m/s are highlighted by decreasing color depth, whereas the stress profiles extrapolated for $\dot{s}\to 0$ is shown in black. See also Table 1 for characteristic properties

The underlying stress profiles *σ*_*zz*_(*s*) of tensile testing the silica, cellulose, and magnetite interfaces to epoxy resin are shown in Fig. 3, respectively. While at least a qualitative comparison of the different strain rates may be related to the runs performed at $\dot{s}$ = 0.015, 0.15, and 1.5 m/s, the limited times of molecular dynamics simulations prevent the direct implementation of tensile testing at the typically much slower rates applied in the experiment. For this reason, we performed additional relaxation analyses that mimic a quasi-static setup, i.e., stress profiles extrapolated to $\dot{s}\to 0$. For selected snapshots (*s* = *s*(*σ*_max_), 1 and 2 nm, respectively), additional runs at constant *s* (pull-and-hold) were performed. On the basis of exponential relaxation, we describe the time-dependent profiles as

1 $${\sigma}_{zz}\left(t;s= const.\right)=A\cdot {e}^{\frac{-t}{\tau }}+{\sigma}_{\dot{s}\to 0}(s)$$

where ${\sigma}_{\dot{s}\to 0}(s)$ describes the residual stress upon infinite relaxation time. In turn, the first term indicates the effect of limited relaxation times. The corresponding fits at *s*(*σ*_max_) lead to *A* = 3.6, 3.6, and 4.5 MPa and *τ* = 3, 1.5, and 7 ns for epoxy interfaces to silica, cellulose, and magnetite, respectively.

To one side, we derived the maximum stress extrapolated for $\dot{s}\to 0$ as also denoted in Table 1. In our earlier study dedicated to the pure epoxy resin, this strategy showed excellent agreement to experimental tensile testing [30]. Comparing the maximum stress experienced for the bulk epoxy resin to those computed for our interface models, we find *σ*_max_ to be reduced by 60–75%. On the other hand, also the maximum elongation before bond dissociation is drastically reduced.

**Table 1:** Comparison of tensile testing runs A (block-wise interface separation) described by $\dot{s}=\infty$ and B (relaxed scans) featuring the theoretical limit of maximum stress and fragmentation energy and data collected from explicit molecular dynamics runs in brackets below. While run A is unphysical for the bulk epoxy resin, also for the interface systems we find block-wise separation as a rather poor approximation to relaxed scans performed by distinct tensile testing at 300 K. In turn, the data we derived from extrapolation to $\dot{s}\to 0$ (highlighted in bold) along the relaxation runs resulted in reliable values compared to bulk epoxy

$\dot{s}=\infty$ / $\dot{s}\to 0$ $\left(\dot{s}=1.5,0.15,0.015\right)$	Silica-epoxy	Cellulose-epoxy	Magnetite-epoxy	Bulk epoxy (ref. [30])
***σ***_***max***_/ *MPa*	885/**33** (49, 43, 39)	872/**21** (35, 29, 26)	1181/**26** (44, 37, 32)	- /**84** (-, -, 142)
$-\boldsymbol{\int}{\boldsymbol{\sigma}}_{\boldsymbol{zz}}\boldsymbol{ds}/\kern0.5em \frac{J}{m^2}$	0.20/**0.018** (0.03, 0.02, 0.02)	0.17/**0.014** (0.02, 0.02, 0.02)	0.22/**0.027** (0.05, 0.04, 0.04)	- /**0.98** (-, -, 1.6)
**s**(***σ***_***max***_)/ *nm*	0.06/**0.19** (0.19, 0.16, 0.19)	0.05/**0.3** (0.31, 0.25, 0.3)	0.04/**0.31** (0.28, 0.27, 0.31)	- /**10** (-, -. 10)
***s***_***sep***_/ *nm*	0.91/**1.1** (1.01, 1.05, 1.1)	0.74/**1.3** (1.3, 1.5, 1.5)	0.76/**2.1** (2.1, 2.3, 2.6)	- /**21** (-, -, 23)

This leads to substantially lower work of separation. For its quantification, we resort to characterizing the stress profiles by a simple quantitative model as recently suggested for the bulk epoxy resin [13]. Therein, a bi-linear fit is suggested to (i) describe the (visco-) elastic deformation regime. For this, we take use of the maximum stress *σ*_max_ as well as the underlying displacement *s*(*σ*_max_) to describe the linear increase in stress within 0 ≤ *s* ≤ *s*(*σ*_max_). In turn, (ii) yielding is modelled for *s*(*σ*_max_) < *s* ≤ *s*_sep_ by assuming a linear decline of the stress until *σ*(*s*_sep_) = 0. While *σ*_max_ and *s*(*σ*_max_) are well-defined properties assessable from the stress profiles (Fig. 3, see also Table 1), we define *s*_sep_ in such manner that the overall work of separation is retained by the bi-linear model, hence using:

2 $$-\int_0^{\infty }{\sigma}_{zz}(s)\ ds=\frac{1}{2}{\sigma}_{max}\cdot s\left({\sigma}_{max}\right)+\frac{1}{2}{\sigma}_{max}\cdot \left[{s}_{sep}-s\left({\sigma}_{max}\right)\right]$$

The resulting values of *s*_sep_ are denoted in Table 1. For illustration, the bi-linear approximation is shown in Fig. 3 using dashed lines.

Comparing *s*_sep_ of the bulk epoxy resin to our interface systems, we find a rather drastic reduction by 90–95%. Combined with the reduction of the maximum stress before fracture/delamination, this leads to diminishing of the work of separation from about 1 J/m^2^ observed for the bulk epoxy resin to only 0.01–0.02 J/m^2^ at the epoxy/cellulose and epoxy/metal oxide interfaces. These properties not only serve as descriptors for the adhesion of epoxy-cellulose/metal oxide interfaces, but also offer atomic-scale input for cohesive zone models [32–34]. On this basis, continuum models may benefit from the fundamental information collected from molecular dynamics simulations to breach out to larger length scales and more complex composite structure.

## Conclusion

We presented a detailed analysis of epoxy resin interactions to interfaces to metal oxide and cellulose surfaces, thus offering molecular scale insights into the foundations of epoxy-based composites. Rather than simply attaching blocks of epoxy and metal oxide or cellulose slabs, we explicitly explored the curing of the epoxy network in the presence of composite constituents. This provides reliable models of atomic detail, from which the interplay of epoxy-linker interactions and the surfaces of cellulose/metal oxide constituents is elaborated in terms of hydrogen bonding and salt-bridges.

The nanometer scale models used in the present study are based on sandwich-type alignment of 2D-periodic layers—which facilitates the direct assessment of stress profiles as functions of interface separation. On this basis, we suggest a bi-linear fit model to describe the work of separation within coarse-grained or continuum models. Indeed, coarsening of the currently atomic models towards micrometer scale resolution appears crucial for addressing particle- and/or fiber-enforces epoxy composites as employed for technical applications. To name an illustrative example, clutch discs used in automobiles are typically based on epoxy composites featuring micrometer-millimeter-sized particles (silica, clay, etc.) and centimeter scale fibers (cellulose, aramid, etc.), respectively [2, 3]. For the simulation of such complex composite systems, we currently develop coarse-grained models that provide the required computational efficiency while reproducing the atomic-scale learnings of the present study.

The current sandwich-type model systems arguably reflect only the first step within a series of studies needed for the comprehensive understanding of particle/fiber-enforced epoxy composites. However, for the somewhat simpler system of steel-epoxy resin interfaces, we suggest the layer-wise contact as a quite realistic model for understanding adhesion. Focusing on magnetite Fe_3_O_4_ rather than pure iron or rusted surfaces, our simulations address machine parts that were pre-treated via bluing processes [35].
